# Iranian healthcare professionals’ knowledge, attitudes, and use of complementary and alternative medicine: a cross sectional study

**DOI:** 10.1186/s12906-021-03421-z

**Published:** 2021-09-30

**Authors:** Alireza Jafari, Mohaddeseh Zanganeh, Zahra Kazemi, Elaheh Lael-Monfared, Hadi Tehrani

**Affiliations:** 1 Department of Health Education and Health Promotion, School of Health, Social Development and Health Promotion Research Center, Gonabad University of Medical Sciences, Gonabad, Iran; 2 Student Research Committee, Torbat Heydariyeh University of Medical Sciences , Torbat Heydariyeh, Iran; 3 Student Research Committee, School of Public Health and Safety , Shahid Beheshti University of Medical Sciences, Tehran, Iran; 4 Department of Health Education and Health Promotion, Social Determinants of Health Research Center, Mashhad University of Medical Sciences, Mashhad, Iran

**Keywords:** CAM, Knowledge, Attitude, Behavior, Health care personnel

## Abstract

**Background:**

The present study aims to investigate the knowledge, attitude, and performance of Iranian Healthcare Professionals (HP) about Complementary and Alternative Medicine (CAM) modalities.

**Methods:**

This cross-sectional study was carried out on 210 HP in 2019. Samples were selected from healthcare centers, clinics, and hospitals using census sampling. Data collection tools included demographic information, attitude, knowledge, and the amount of use of CAM modalities. Data analysis was performed using SPSS ver. 24.

**Results:**

In this study, the response rate was 85.3% (*n*=209). A majority of respondents had a positive attitude toward CAM (*n*=166, 79%), but their level of knowledge was limited (*n*=154, 73.6%). The most commonly used CAM modalities were herbal medicine (93.2%), exercise therapy (75.4%), and hydrotherapy (75.2%), respectively, and the least commonly used ones were magnetic therapy (2.9%) and hypnosis (4.8%). The most important reasons for the use of CAM modalities by HP included fewer side effects than medical treatments (57.4%), its lowest cost than medical treatments (34.9%), non-serious disease with no need for referral to a clinic (32.1%), and its more convenient access than medical treatments (30.6%). The results showed that there was a significant relationship between the education level and the use of CAM modalities (*p*<0.05). There was also a significant relationship between the suggestion of CAM modalities and the amount of use of these modalities, and those who used these modalities would also have recommended them to their clients more frequently (*p*<0.05).

**Conclusion:**

The results showed that most of HP used at least one of the CAM modalities and had a positive attitude towards CAM. As the level of knowledge was limited, training courses should be implemented to increase health practitioner’s level of knowledge on CAM.

**Supplementary Information:**

The online version contains supplementary material available at 10.1186/s12906-021-03421-z.

## Background

In recent years, Complementary and Alternative Medicine (CAM) has been used more commonly, along with conventional medical treatments among patients and individuals [1–3]. CAM is defined as a group of diverse medical and healthcare systems, practices, and products, which are currently not considered to be an integral part of conventional medicine [4]. Complementary medicine “refers to the therapies that complement conventional medicine (or allopathic) medicine and are used together with conventional medicine, and alternative medicine is used instead of conventional medicine”. In this regard, alternative medicine “refers to those therapeutic approaches taken instead of traditional medicine and used to treat or ameliorate disease” [5].

In a systematic review, the rate of CAM use in the United States increased from 36% in 2002 to 38% in 2007 [6]. In the United Kingdom about 41% of communities and patients had used CAM last year, and 51% of them had used CAM during their lifetime [7]. About 26% of Europeans use CAM modalities [8]. Also, the results of another study showed that the usage rate of CAM was 26.4%, ranging from less than 10% in Slovenia, Poland and Bulgaria to more than 50% in the Republic of Korea, China and the Philippines [9]. Healthcare Professionals (HP) are more likely to use CAM compared to other community members and other occupations [10]. The results of a meta-analysis showed that nurses need training courses to obtain the required information about CAM [11]. Also, the results of a study showed that physicians had more positive attitudes about CAM in 2012 than in 2004. In 2004, only 44% and in 2012 about 71% of physicians were willing to refer their patients to CAM specialists [12].

A study conducted on Iranian patients showed that 53.3% of them used CAM, and they had insufficient information on its benefits, risks, and side effects, which could put them at risk [13]. About 95% of Iranian patients with cancer also used at least one of the CAM modalities [14]. In addition, the results show that the use of CAM in Iranian patients has also increased from 26% in 2009 to 67% in 2015 [15, 16]. 31.3 % of dermatology outpatients have used at least one of the CAM therapies [17]. In Iran about 86.6% of health care providers used CAM modalities [18]. A study on Iranian nurses showed that their knowledge of CAM is low and most of them believed that the use of CAM modalities is useful for the treatment of diseases, and they had a positive attitude towards it [19].

HP’s knowledge of CAM is limited, so most of them believe that obtaining this information is essential [20, 21]. Most of Iranian nurses have a positive attitude towards the use of CAM, but they have insufficient knowledge toward CAM [22]. Also, patients often obtain information about CAM from informal sources (such as friends, family, the Internet, and the media), which may bring them wrong information and put them at risk [23–25].

HP keeps in touch with people during the first stage of prevention and can provide their clients with useful and reliable information about CAM. Since these patients may use CAM modalities to treat their disease, in addition to conventional medical treatments, HP’s knowledge of the nature and effect of CAM can help in the proper use of that and also in obtaining accurate information by patients and community members. So, the aim of this study was to investigate the knowledge, attitude, and performance of healthcare professionals about CAM modalities in Iran, and whether their CAM use affected their recommendations of these modalities to patients.

## Methods

### Sampling and participants

**Fig. 1:**
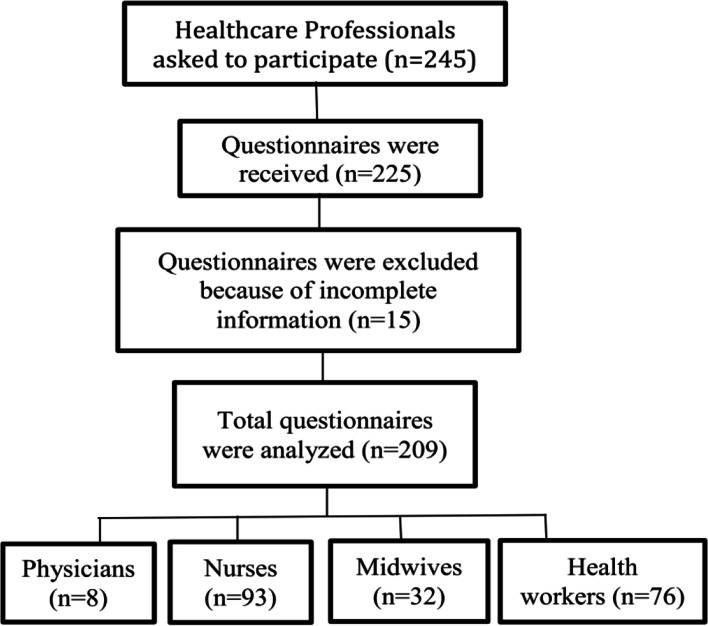
Flowchart of selecting participants in this study

This research was a cross sectional-analytical study conducted on HP in Torbat Heydariyeh city, Iran, from November 2018 to April 2019. The participants were selected using consensus sampling from eight health care centers, two hospitals, and two clinics. The questionnaire was distributed to all HP (*n* = 245), and 225 questionnaires were received. In this study, 15 questionnaires were excluded due to incomplete information, and 209 questionnaires were finally analyzed. Participants in this study included 8 physicians, 93 nurses, 32 midwives and 76 health workers (Fig. 1).

### Data collection

The researchers distributed the questionnaire in paper form to healthcare professionals and completed it through self-reporting. After obtaining the necessary approval from the ethics committee, the research process began. For data collection, the researcher referred to healthcare centers, clinics, and hospitals. Before starting the study, the study objectives were explained to the participants and informed consent was obtained. Moreover, they assure them that their information will be kept secret from the research team. The inclusion criteria include that the person is working in the health system (including health centers, clinics, or hospitals) and is willing to participate in the study. The exclusion criterion was incomplete questionnaire information.

### Measures

The content of the questionnaire is divided into four parts: demographic, CAM practice, attitude, knowledge, and use of CAM.

#### Demographic section

This part includes questions such as age, sex, education level, occupation, and marital status.

#### CAM practice

This part includes questions such as suggesting CAM modalities to clients, asking questions from clients about CAM modalities, predicting the future status of CAM modalities, the reason for using of CAM modalities, and source of information about CAM modalities (Additional file 1).

#### Attitude section

A standard questionnaire was used to assess the people’ level of attitude about CAM modalities. This part consists of 18 questions, which were scored using a five-point Likert scale (strongly agree, agree, neither agree or disagree, disagree, and strongly disagree) and assessed the individual's attitude toward CAM modalities. In this section, the lowest total score and the highest total score are 18 and 90, respectively. This section includes three subscales of health believes contains 8 questions (questions 1 to 8, lowest score=8, highest score=40), the effect of CAM contains 5 questions (questions 9 to 13, lowest score=5, highest score=25), and risk of CAM contains 5 questions (questions 14 to 18, lowest score=5, highest score=25). The Shorofi study evaluated the validity and reliability of this questionnaire and its Cronbach's alpha was 0.929 [26] (Additional file 2).

#### Knowledge section

A researcher-made questionnaire was used to assessment the participants’ level of knowledge about 17 commonly used CAM modalities in Iran (acupuncture, music therapy, energy therapy, hypnosis, massage therapy, magnetic therapy, meditation/relaxation, yoga, therapeutic exercise, leech therapy, bloodletting, therapeutic touch, hydrotherapy, pressure therapy, vitamin supplements, herbal medicine, nutritional therapy). This section includes two subscales of mind- body practices contains 14 modalities (lowest score=14, highest score=70) and natural product contains 3 modalities (lowest score=3, highest score=15). The scoring scale of this section was used a 5-point Likert scale (low, moderate, good, very good, don’t know) with a sample question: how much do you know about acupuncture? In this section, the lowest total score and the highest total score are 17 and 85, respectively (Additional file 3).

#### Use of CAM section

This section checks whether participants use the 17 CAM modalities (daily, weekly, monthly, use CAM when needed, and do not use any CAM modalities). The 17 CAM modalities were selected based on the two standard classifications adopted by the National Center for Complementary and Integrative Health (NCCIH )[27] (Additional file 4).

### Validity and reliability

The face validity of the questionnaire was evaluated by providing a questionnaire to an expert panel of 9 health education experts and CAM specialists. The reliability of the questionnaire was evaluated by 10 participants (1 physician, 3 nurses, 2 midwives, and 4 health care workers), and Cronbach's alpha of attitude and knowledge sections were 0.70 and 0.91, respectively.

### Statistical analyses

Data analysis was then carried out using SPSS ver. 24. To describe the data, the relevant tables, and charts, the number and percentage were used for qualitative variables, and the mean and standard deviation were used for quantitative variables. Data analysis was also carried out using of Chi-square test at significant level of less than 0.05.

## Results

### Socio-demographic characteristics

The present study was carried out on 210 HP in Iran. The response rate for this study was 85.3%. The mean (± standard deviation) of participants’ age was 29.71 (± 7.58) years. Of the participants, 27.4% were men, and 72.6% were women. Most participants were married (74.4%) and had undergraduate education (71.5%). The participants in this study included physicians (3.8%), nurses (45.5%), midwives (15.3%), and health workers (36.4%). Most of them also had less than five years of work experience. There was a significant relationship between the level of education and the participants’ use of CAM (*p*=0.015). This means that participants with a bachelor’s degree used more CAM (*p*=0.015).

### CAM practice

Most of the HP (61.7%) asked their clients about CAM, and most of them (64.2%) had also suggested these modalities to their clients. About the future status of CAM, 67% of participants believed that CAM will be prescribed together with conventional medical treatments (Table 1). There was also a significant relationship between the recommendation of CAM to patients and the participant’s use of these modalities. Meaning that participants who used CAM also suggested these modalities to their patients (*p*=0.045) (Table 1). In this study, most HP obtained information about CAM from the internet (65.6%), specialists (50.7%), friends (39.2%) and booklets (24.4 %).

**Table 1:** Demographic characteristics and their relation with the use of CAM among healthcare professionals

Variables		Data (***N*** = 210)	Use of CAM (at least once during lifetime)		
			Yes	No	***P***-value*
		n (%)	n (%)	n (%)	
**Sex**	Men	57 (27.4)	55 (96.5)	2 (3.5)	0.183
	Women	151 (72.6)	150 (99.3)	1 (0.7)	
**Marital status**	Marriage	151 (74.4)	149 (98.7)	2 (1.3)	1
	Single	52 (25.6)	51 (98.1)	1 (1.9)	
**Type of healthcare professionals**	Physicians	8 (3.8)	7 (87.5)	1 (12.5)	0.036
	Nurses	93 (45.5)	91 (97.8)	2 (2.2)	
	Midwives	32 (15.3)	32 (100)	0	
	Health workers	76 (36.4)	7 (100)	0	
**Years of practice**	5 ≥	123 (61.2)	121 (98.4)	2 (1.6	0.989
	> 5	78 (38.8)	77 (98.7)	1 (1.3)	
**Recommending complementary / alternative therapies to your clients**	Yes	131 (64.2)	131 (100)	0	0.045
	No	73 (35.8)	70 (95.9)	3 (4.1)	
**Do you have any questions from your clients about their use of complementary / alternative therapies?**	Yes	124 (61.7)	124 (100)	0	0.055
	No	77 (38.3)	74 (96.1)	3 (3.9)	
**How do you think the status of alternative therapies / therapies in the future?**	Replace medical treatments	11 (5.3)	11 (100)	0	0.957
	Both medical treatments and complementary therapies will be prescribed	139 (67.1)	137 (98.6)	2 (1.4)	
	Only medical treatments will be prescribed	5 (2.4)	5 (100)	0	
	I do not know	52 (25.1)	51 (98.1)	1 (1.9)	

* Chi-square

### Attitudes toward CAM

In this study, 79% (*n* = 166) of the respondents had a positive attitude towards CAM. Based on the results in Table 2, the majority of participants (89.9%) believed that both body and mind must be treated to restore complete health to patients. Most of the participants (60%) believed that CAM therapies could be used as a complement to the treatments used in conventional health care. Also, the majority of the participants (69.2%) believed that patients did not have sufficient knowledge of CAM in the hospital, and only 3.4% of participants disagreed (Table 2).

**Table 2:** Attitude of healthcare professionals towards CAM

Items		Level of agreement n (%)					Subscales
							Mean (Standard deviation)
		Strongly disagree	Disagree	Unsure	Agree	Strongly agree	
**Health believes/philosophical view**	1. CAM is an important aspect of my own family’s health care	11 (5.3)	22 (10.6)	93 (44.9)	73 (35.3)	8 (3.9)	25.73 (3.47)
	2. Both mind and body must be treated for the patient to regain complete health	3 (1.4)	4 (1.9)	14 (6.7)	114 (54.5)	74 (35.4)	
	3. Patients should have the right to choose between conventional treatments and CAM therapies in health care	5 (2.4)	19 (9.2)	30 (14.5)	127 (61.4)	26 (12.6)	
	4. Conventional health care services are too impersonal	5 (2.5)	43 (21.1)	79 (38.7)	62 (30.4)	15 (7.4)	
	5. People are afraid of examinations and treatments from conventional health care services	13 (6.3)	70 (33.8)	61 (29.5)	57 (27.5)	6 (2.9)	
	6. Conventional health care services do not meet people’s expectations	14 (6.8)	54 (26.3)	60 (29.3)	68 (33.2)	9 (4.4)	
	7. The changes that have taken place in the conventional health care system have encouraged people to use CAM methods to a greater extent	6 (2.9)	33 (16)	80 (38.8)	81 (39.3)	6 (2.9)	
	8. Patients with an untreatable condition should be encouraged to seek CAM therapies	6 (2.9)	27 (13.1)	76 (36.9)	72 (35)	25 (12.1)	
**Effect of CAM**	9. Some forms of CAM therapies are as effective as conventional treatments	5 (2.4)	27 (13)	81 (39.1)	83 (40.1)	11 (5.3)	14.91 (2.01)
	10. Surgical patients can be helped by using CAM therapies	7 (3.5)	19 (9.5)	87 (43.5)	77 (38.5)	10 (5)	
	11. Some forms of CAM therapies work better than conventional treatments	6 (2.9)	30 (14.6)	89 (43.2)	69 (33.5)	12 (5.8)	
	12. Positive effects of CAM therapies are in most cases due to placebo effect	7 (3.4)	27 (13.1)	120 (58.3)	50 (24.3)	2 (1)	
	13. CAM therapies could be used as a complement to the treatments used in conventional health care	4 (2)	25 (12.2)	53 (25.9)	100 (48.8)	23 (11.2)	
**Risk of CAM**	14. Patients are not adequately informed about CAM therapies in the hospital	7 (3.4)	15 (7.2)	42 (20.2)	110 (52.9)	34 (16.3)	15.28 (2.34)
	15. CAM therapies are completely safe	9 (4.3)	50 (24)	100 (48.1)	36 (17.3)	13 (6.3)	
	16. The use of CAM therapies may delay the patients’ decision to contact conventional health professionals	6 (2.9)	13 (6.3)	65 (31.4)	117 (56.5)	6 (2.9)	
	17. CAM therapies may involve unknown risk factors for users’ health	6 (2.9)	25 (12.3)	80 (39.2)	81 (39.7)	12 (5.9)	
	18. CAM therapies are offered just for financial gain by quack health careers	17 (8.2)	70 (33.8)	84 (40.6)	31 (15)	5 (2.4)	

### Knowledge toward CAM

In this study, most respondents (*n* = 154, 73.6%) had limited knowledge of CAM modalities. Based on the results, most of the HP's knowledge about CAM modalities was related to exercise therapy (51.7 %), Herbal medicine (51.7 %), vitamin supplements (51.4 %), Nutritional therapy (46.9 %), Music therapy (28.4 %), Bloodletting (27.6 %), Magnetic therapy (24.4 %), and Hydrotherapy (24.1%), respectively (Table 3).

**Table 3:** Recommendation of CAM modalities to patients and CAM knowledge among the healthcare professionals

CAM modalities		Recommendation CAM modalities to clients		All modalities		Knowledge					Subscales
		Yes	No	Yes	No	I do not know	Low	Medium	Good	Very good	Mean (Standard deviation)
		n(%)	n(%)	n(%)	n(%)	n(%)	n(%)	n(%)	n(%)	n(%)	
**Mind and body practices**	1. Acupuncture	26 (12.4)	183 (87.6)	185 (88.5)	24 (11.5)	44 (21.1)	68 (32.5)	66 (31.6)	25 (12)	6 (2.9)	33.49 (±10.78)
	2. Music therapy	55 (26.4)	153 (73.6)			39 (18.8)	52 (25)	58 (27.9)	46 (22.1)	13 (6.3)	
	3. Energy therapy	11 (5.3)	198 (94.7)			52 (24.9)	88 (42.1)	44 (21.1)	17 (8.1)	8 (3.8)	
	4. Hypnosis	5 (2.4)	204 (97.6)			52 (25)	101 (48.6)	39 (18.8)	8 (3.8)	8 (3.8)	
	5. Massage therapy	41 (19.7)	166 (79.8)			50 (23.9)	56 (26.8)	52 (24.9)	36 (17.2)	15 (7.2)	
	6. Magnetic therapy	1 (0.5)	208 (99.5)			79 (38)	104 (50)	20 (9.6)	3 (1.4)	2 (1)	
	7. Meditation/Relaxation	17 (8.1)	192 (91.9)			62 (30.1)	80 (38.8)	40 (19.4)	14 (6.8)	10 (4.9)	
	8. Yoga	13 (6.2)	196 (93.6)			57 (27.7)	76 (36.9)	46 (22.3)	19 (9.2)	8 (3.9)	
	9. Exercise therapy	79 (37.8)	130 (62.2)			17 (8.2)	25 (12.1)	58 (28	67 (32.4)	40 (19.3)	
	10. Leech therapy	22 (10.5)	187 (89.5)			48 (23)	69 (33)	55 (26.3)	23 (11)	14 (6.7)	
	11. Bloodletting	40 (19.1)	169 (80.9)			31 (15)	55 (26.6)	64 (30.9)	38 (18.4)	19 (9.2)	
	12. Therapeutic touch	7 (3.3)	202 (96.7)			72 (35)	80 (38.8)	32 (15.5)	11 (5.3)	11 (5.3)	
	13. Hydrotherapy	80 (38.3)	129 (61.7)			44 (21.2)	61 (29.3)	53 (25.5)	39 (18.8)	11 (5.3)	
	14. Pressure therapy	27 (13)	181 (87)			76 (36.7)	77 (37.2)	26 (12.6)	15 (7.2)	13 (6.3)	
**Natural products**	15. Vitamin supplements	107 (51.2)	101 (48.3)	169 (80.9)	40 (19.1)	15 (7.2)	25 (12)	61 (29.3)	62 (29.8)	45 (21.6)	10.11 (±2.63)
	16. Herbal medicine	119 (56.9)	88 (42.1)			8 (3.9)	24 (11.6)	68 (32.9)	78 (37.7)	29 (14)	
	17. Nutritional therapy	80 (38.3)	129 (61.7)			17 (8.1)	31 (14.8)	63 (30.1)	82 (39.2)	16 (7.7)	

### CAM recommendation to clients

Most of the CAM modalities suggested by HP to the patients were herbal medicine, vitamin supplements, nutrition therapy, and hydrotherapy (Table 3).

### Personal use of CAM

**Fig. 2:**
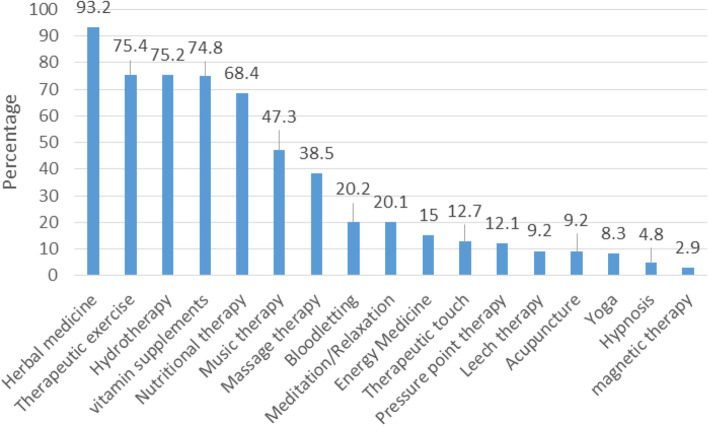
CAM modalities use by healthcare professionals at least once during lifetime

All HP reported that they had used at least one of the modalities of mind and body practices and natural products. The most commonly used CAM modalities were herbal medicine (such as Mint, Rosemary, Ginger, Chamomile, Thyme) (93.2%), exercise therapy (75.4%), hydrotherapy (75.2%), and vitamin supplements (74.8%) (Table 4). Also, the least commonly used CAM modalities were also magnetic therapy (2.9%), hypnosis (4.8%), yoga (8.3%), acupuncture (9.2%), and leech therapy (9.2%) (Table 4, Fig. 2).

**Table 4:** The frequency of CAM modalities uses by healthcare professionals

CAM modalities		n(%)				
		Daily	Weekly	Monthly	I use if necessary	I have not used it yet
**Mind and body practices**	1. Acupuncture	1 (0.5)	-	-	18 (8.7)	188 (90.8)
	2. Music therapy	33 (15.9)	14 (6.8)	2 (1)	49 (23.7)	52.7
	3. Energy therapy	4 (1.9)	4 (1.9)	2 (1)	21 (10.1)	176 (85)
	4. Hypnosis	1 (0.5)	-	-	9 (4.3)	198 (95.2)
	5. Massage therapy	5 (2.4)	6 (2.9)	8 (3.8)	61 (29.3)	128 (61.5)
	6. Magnetic therapy	-	-	1 (0.5)	5 (2.4)	201 (97.1)
	7. Meditation/Relaxation	6 (2.9)	2 (1)	8 (3.8)	26 (12.4)	167 (79.9)
	8. Yoga	-	-	2 (1)	15 (7.3)	91.7
	9. Exercise therapy	28 (13.5)	40 (19.3)	16 (7.7)	72 (34.8)	51 (24.6)
	10. Leech therapy	-	2 (1)	2 (1)	15 (7.2)	188 (90.8)
	11. Bloodletting	2 (1)	-	3 (1.4)	37 (17.8)	166 (79.8)
	12. Therapeutic touch	2 (1)	5 (2.4)	1 (0.5)	18 (8.7)	181 (87.4)
	13. Hydrotherapy	4 (1.9)	8 (3.8)	8 (3.8)	50 (24)	138 (66.3)
	14. Pressure therapy	5 (2.4)	2 (1)	2 (1)	16 (7.7)	182 (87.5)
**Natural products**	15. Vitamin supplements	18 (8.7)	14 (6.8)	28 (13.6)	94 (45.6)	52 (25.2)
	16. Herbal medicine	19 (9.2)	16 (7.7)	28 (13.5)	130 (62.8)	14 (6.8)
	17. Nutritional therapy	13 (6.3)	10 (4.9)	23 (11.2)	95 (46.1)	65 (31.6)

### Reasons for Using CAM

The most important reasons for HP's use of CAM were fewer side effects than medical treatments (57.4%), its lowest cost than medical treatments (34.9%), non-serious disease with no need for referral to a clinic (32.1%), and its more convenient access than medical treatments (30.6%) (Table 5).

**Table 5:** Reasons for the use of CAM modalities by healthcare professionals

Items	Yes		No	
	n	%	n	%
1. Its lowest cost than medical treatments	73	34.9	136	65.1
2. Dissatisfaction with medical treatment	32	15.3	177	84.7
3. Recommended by a physician	26	12.4	183	87.6
4. Its more convenient access than medical treatments	64	30.6	145	69.4
5. To strengthen the effects of the medications prescribed by a physician	32	15.3	177	84.7
6. Fewer side effects than medical treatments (medication Chemical)	120	57.4	89	42.6
7. Because it has better effectiveness than medical treatments (drugs Chemical)	36	17.2	173	82.8
8. To control my disease	27	12.9	182	87.1
9. Problem is not serious enough to go to a clinic	67	32.1	142	67.9
10. Advice from family or friends	71	34	136	64.1
11. The effectiveness has been proven to me	62	29.7	146	69.9
12. It will improve my health	36	17.2	171	81.8
13. Because it fits with my lifestyle	48	23.1	158	76

## Discussion

The aim of this study was to investigate the knowledge, attitude, and performance of healthcare professionals about CAM modalities in Iran, and whether their CAM use affected their recommendations of these modalities to patients. The results of this study showed that HP had a positive attitudes towards CAM, and most of them used CAM. Also, HP had an insufficient knowledge about CAM modalities.

### Personal use of CAM

The results of the current study showed that most of the HP used at least one CAM modalities, and the most commonly used modalities were herbal medicine, exercise therapy, hydrotherapy, and vitamin supplements, respectively. The results of a study on HP in Trinidad and Tobago showed that most of the HP used CAM [20]. Moreover, a study on HP in the United States showed that about 76% of them used at least one of the CAM modalities [10]. In a study on nurses, Gyasi stated that the most commonly used modalities including non-herbal supplements, relaxation techniques, massage therapy, spirituality/prayer therapy, and music therapy, and those who obtained higher knowledge scores, used CAM modalities more frequently [23].

A systematic review study showed that the usage rate of CAM ranges from 25 to 96% in nurses. A study in Nigeria showed that the most commonly used modalities of CAM in HP were worship and prayer [28]. Also, in another study, the most commonly used modalities by HP were massage therapy, non-herbal supplements, and music therapy [27]. The variety of modalities used or the different rates of using CAM in studies may be due to the different cultural and religious or geographic regions of each community.

### Attitudes toward CAM

The results of this study showed that only 21% of the participants had negative attitudes, while most of them had positive attitudes toward CAM. A systematic review study on nurses showed that most of them had positive attitudes toward the use of CAM [11]. The results of another study on HP also showed that most of them had positive attitudes toward CAM [22]. Another study conducted by Nejatian in Iran showed that health care providers had positive attitudes toward use of CAM [18]. In a study by Shorofi, about 60% of nurses had positive attitudes toward CAM [26]. The results of Aveni’s study also showed that most healthcare professionals have a positive attitude towards CAM and believe that the use of CAM could be useful for the treatment of patients [29].

### Knowledge toward CAM

The results of the present study show that most of the participants had inadequate level of knowledge about CAM. The first step in performing any behavior is to acquire the necessary knowledge. Lack of sufficient knowledge may endanger a people’s health or others health. Low knowledge on CAM can have adverse consequences for those HP who recommend using these modalities to their clients [23]. Therefore, due to the lack of sufficient knowledge of healthcare professionals, it is necessary to provide some appropriate training courses to improve their knowledge toward CAM modalities.

The results of a study showed that, those HP who had inadequate knowledge about CAM were not sure that these modalities are useful to their clients and that they were skeptical about whether to suggest these modalities to the clients [30]. A study conducted by Gyasi in Ghana showed that nurses had limited knowledge of CAM, and only 4.5% of nurses had a high level of knowledge about CAM. Nurses also reported that they had an excellent knowledge on spirituality/prayer therapy, massage therapy, relaxation modalities, non-herbal supplements, and music therapy [23]. In the Stub’s study, most of the personnel information was related to acupuncture, massage, and mindfulness [31]. In a national study performed in Sweden, Bjerså showed that the majority of HP had limited knowledge on CAM (95.7%) [21]. The results of Aveni’s study on healthcare professionals showed that most of them do not sufficient knowledge on CAM, and most of them also believe that healthcare professionals should be able to inform patients about CAM [32].

### The source of information about CAM

The results show that most of the HP get information on the CAM from the internet, specialists, and friends. People can access various information sources to perform their behaviors, and they can also obtain information from different sources. However, it should be pointed out that all information resources may not be reliable and people may obtain incorrect information and expose themselves to various risks. Currently, the Internet is one of the most accessible sources of information. Given that the content on the Internet is very extensive and all content is untrustworthy, it is essential that health professionals have the necessary knowledge or a reliable CAM science website. Therefore, they can finally obtain accurate information on CAM and provide appropriate information to their clients.

A systematic review study showed that HP obtained their information on CAM from professional colleagues, magazines, and the internet [11]. The results of the Stub’s study showed that most medical doctors gather information about complementary cancer modalities from some guidelines [33]. A study conducted by El-Olemy in Egypt showed that HP obtained most of the information related to CAM from the media, friends and relatives [34]. A systematic review among pharmacy students showed that they refer to the media, CAM specialists, and books to obtain information about CAM [35]. Also, Cooke’s study showed that about 80% of nurses obtain information about CAM through the Internet [36].

### Reasons for using CAM

The most important reasons for the use of CAM by HP were fewer side effects compared to medical treatments, it’s cheaper than conventional treatments. The results of the Anbari’s study showed that the most important reason for using CAM is the concern about the side effects of medical treatments [37]. Based on the results of the Johnson’s study conducted on HP in the United States, the main reason for the use of CAM was general health improvement [10]. Also, the Jane-lovena’s study reported being natural and maintaining a healthy state as the most important reasons for using CAM [38]. There are many reasons why people often use CAM, for example, the effects of these modalities and the increase in conventional medical practice, the treatment of certain diseases, avoiding certain adverse side effects and relatively low costs [39]. People usually use CAM for various reasons, depending on the level of education, place of residence, and socioeconomic and cultural status of each individual.

### Asking questions about CAM from clients by HP

In this study, most of the HP asked questions from their clients about CAM, and most of them had then suggested these modalities to their clients. Education is not a one-way process, but is considered as a two-way learning process, in which information is collected and then shared by HP to patient/client, and the patient/client makes the final decision to perform the behavior. Therefore, in the first step, HP should ask the necessary questions from their clients, and then they provide them with appropriate counseling [30, 40].

### CAM recommendation to clients

In this study, HP noted that they more frequently suggested the use of herbal medicine, vitamin supplements, nutrition therapy, and hydrotherapy to their clients. Also, there was a significant relationship between CAM recommendations and the use of these modalities, and HP who uses these modalities will recommend them to their clients. Moreover, the evaluation of HP showed that they had suggested the use of massage, acupuncture, and pressure therapy modalities to their clients [21]. In addition, the results of the study showed that only a small number (26%) of the personnel suggested the use of CAM to their clients [20]. In this regard, the results of a survey showed that the majority of nurses (87%) recommend the use of at least one CAM modalities for patients [23]. Also, the results of a Hall's study showed that 50% of nurses recommend the use of CAM for surgical patients [26]. The results of the study showed that specialists who do not have sufficient knowledge of CAM doubt whether these modalities will be offered to their clients [30].

The difference in the amount of CAM suggested to clients in the other studies could be due to the different levels of attitude, knowledge, and use of CAM in HP. Given that most HP used CAM in this study and had a positive attitude toward CAM, it is predictable to recommend these modalities to their clients.

### Predict the state of CAM in the future

Regarding the future use of CAM, 67% of HP believe that CAM will be prescribed along with conventional medical practices. In addition, the results of similar studies indicated that HP believes that CAM will be integrated into conventional medical practices and may be prescribed in the future [20, 41].

## Limitation

One of the limitations of this study was that not all HP cooperated. Another limitation of this study was that the information was self-reported.

## Implications

Based on the results of this study, it is it is recommended to conduct similar studies on patients and clients in health centers.

## Conclusion

The results of the current study showed that the majority of HP used at least one of the CAM modalities during their lifetime. In this study, HP had a positive attitude toward CAM, while they had limited knowledge of these modalities. The results of various studies indicated that the use of CAM in patients is increasing. Therefore, it is necessary that HP as a reliable source for health information, take part in education courses on CAM to obtain the relevant information for providing useful counseling for clients.

## Supplementary Information

**Additional file 1.** Questionnaire of CAM practice and reason for using of CAM modalities.

**Additional file 2.** Questionnaire of Attitude towards CAM modalities.

**Additional file 3.** Questionnaire of Knowledge towards CAM modalities.

**Additional file 4.** Questionnaire of using of CAM modalities.

## Data Availability

The data sets used and/or analyzed during the current study were available from the corresponding author on reasonable request.

## Abbreviations

CAM: Complementary and Alternative Medicine
HP: Healthcare Professionals
