# Supplementary material for: Iranian healthcare professionals’ knowledge, attitudes, and use of complementary and alternative medicine: a cross sectional study
Source: BMC Complement Med Ther. 2021 Sep 30;21:244. doi: 10.1186/s12906-021-03421-z (PMC8485522; doi:10.1186/s12906-021-03421-z)
Supplement: Supplementary file 1 — Additional file 1. Questionnaire of CAM practice and reason for using of CAM modalities. [file 12906_2021_3421_MOESM1_ESM.docx]

**Additional files**

**File name:** *Additional file 1*

**Title:** *Questionnaire of CAM practice and reason for using of CAM modalities*

- **Please express your opinion about each of the following statements**

| **Items** | | |
| --- | --- | --- |
| **Recommending complementary / alternative therapies to your clients** | Yes |  |
|  | No |  |
| **Do you have any questions from your clients about their use of complementary / alternative therapies?** | Yes |  |
|  | No |  |
| **How do you think the status of alternative therapies / therapies in the future?** | Replace medical treatments |  |
|  | Both medical treatments and complementary therapies will be prescribed |  |
|  | Only medical treatments will be prescribed |  |
|  | I do not know |  |
| **How do you get Information related to CAM modalities?** | Physician or health care personnel |  |
|  | Newspaper, magazine |  |
|  | Internet |  |
|  | Booklets, pamphlets, brochures |  |
|  | Radio and TV |  |
|  | Asking friends |  |
|  | Satellite channels |  |
|  | I do not know |  |

- **Please express your opinion about the reason for using each of CAM modalities**

| **Items** | **Yes** | **No** |
| --- | --- | --- |
| 1. Its lowest cost than medical treatments |  |  |
| 1. Dissatisfaction with medical treatment |  |  |
| 1. Recommended by a physician |  |  |
| 1. Its more convenient access than medical treatments |  |  |
| 1. To strengthen the effects of the medications prescribed by a physician |  |  |
| 1. Fewer side effects than medical treatments (medication Chemical) |  |  |
| 1. Because it has better effectiveness than medical treatments (drugs Chemical) |  |  |
| 1. To control my disease |  |  |
| 1. Problem is not serious enough to go to a clinic |  |  |
| 1. Advice from family or friends |  |  |
| 1. The effectiveness has been proven to me |  |  |
| 1. It will improve my health |  |  |
| 1. Because it fits with my lifestyle |  |  |
