# Supplementary material for: Iranian healthcare professionals’ knowledge, attitudes, and use of complementary and alternative medicine: a cross sectional study
Source: BMC Complement Med Ther. 2021 Sep 30;21:244. doi: 10.1186/s12906-021-03421-z (PMC8485522; doi:10.1186/s12906-021-03421-z)
Supplement: Supplementary file 2 — Additional file 2. Questionnaire of Attitude towards CAM modalities. [file 12906_2021_3421_MOESM2_ESM.docx]

**Additional files**

**File name:** *Additional file 2*

**Title:** *Questionnaire of Attitude towards CAM modalities*

- **Please express your opinion about each of the following statements**

| **Items** | | **Strongly disagree** | **Disagree** | **Unsure** | **Agree** | **Strongly agree** |
| --- | --- | --- | --- | --- | --- | --- |
| **Health believes/**  **philosophical view** | 1. CAM is an important aspect of my own family’s health care |  |  |  |  |  |
|  | 1. Both mind and body must be treated for the patient to regain complete health |  |  |  |  |  |
|  | 1. Patients should have the right to choose between conventional treatments and CAM therapies in health care |  |  |  |  |  |
|  | 1. Conventional health care services are too impersonal |  |  |  |  |  |
|  | 1. People are afraid of examinations and treatments from conventional health care services |  |  |  |  |  |
|  | 1. Conventional health care services do not meet people’s expectations |  |  |  |  |  |
|  | 1. The changes that have taken place in the conventional health care system have encouraged people to use CAM methods to a greater extent |  |  |  |  |  |
|  | 1. Patients with an untreatable condition should be encouraged to seek CAM therapies |  |  |  |  |  |
| **Effect of CAM** | 1. Some forms of CAM therapies are as effective as conventional treatments |  |  |  |  |  |
|  | 1. Surgical patients can be helped by using CAM therapies |  |  |  |  |  |
|  | 1. Some forms of CAM therapies work better than conventional treatments |  |  |  |  |  |
|  | 1. Positive effects of CAM therapies are in most cases due to placebo effect |  |  |  |  |  |
|  | 1. CAM therapies could be used as a complement to the treatments used in conventional health care |  |  |  |  |  |
| **Risk of CAM** | 1. Patients are not adequately informed about CAM therapies in the hospital |  |  |  |  |  |
|  | 1. CAM therapies are completely safe |  |  |  |  |  |
|  | 1. The use of CAM therapies may delay the patients’ decision to contact conventional health professionals |  |  |  |  |  |
|  | 1. CAM therapies may involve unknown risk factors for users’ health |  |  |  |  |  |
|  | 1. CAM therapies are offered just for financial gain by quack health careers |  |  |  |  |  |
