# Supplementary material for: Iranian healthcare professionals’ knowledge, attitudes, and use of complementary and alternative medicine: a cross sectional study
Source: BMC Complement Med Ther. 2021 Sep 30;21:244. doi: 10.1186/s12906-021-03421-z (PMC8485522; doi:10.1186/s12906-021-03421-z)
Supplement: Supplementary file 3 — Additional file 3. Questionnaire of Knowledge towards CAM modalities. [file 12906_2021_3421_MOESM3_ESM.docx]

**Additional files**

**File name:** *Additional file 3*

**Title:** *Questionnaire of Knowledge towards CAM* *modalities*

- **Please express your opinion about each of the following statements**

| **Items** | | **I do not know** | **Low** | **Medium** | **Good** | **Very good** |
| --- | --- | --- | --- | --- | --- | --- |
| **Mind and Body**  **Practices** | 1. How much do you know about ***acupuncture***? |  |  |  |  |  |
|  | 1. How much do you know about ***Music therapy***? |  |  |  |  |  |
|  | 1. How much do you know about ***Energy therapy***? |  |  |  |  |  |
|  | 1. How much do you know about ***Hypnosis***? |  |  |  |  |  |
|  | 1. How much do you know about ***Massage*** ***therapy***? |  |  |  |  |  |
|  | 1. How much do you know about ***Magnetic*** ***therapy***? |  |  |  |  |  |
|  | 1. How much do you know about ***Meditation/Relaxation***? |  |  |  |  |  |
|  | 1. How much do you know about ***Yoga***? |  |  |  |  |  |
|  | 1. How much do you know about ***Therapeutic*** ***exercise***? |  |  |  |  |  |
|  | 1. How much do you know about ***Leech therapy***? |  |  |  |  |  |
|  | 1. How much do you know about ***Bloodletting***? |  |  |  |  |  |
|  | 1. How much do you know about ***Therapeutic touch***? |  |  |  |  |  |
|  | 1. How much do you know about ***Hydrotherapy***? |  |  |  |  |  |
|  | 1. How much do you know about ***Pressure therapy***? |  |  |  |  |  |
| **Natural**  **Products** | 1. How much do you know about ***Vitamin supplements***? |  |  |  |  |  |
|  | 1. How much do you know about ***Herbal medicine***? |  |  |  |  |  |
|  | 1. How much do you know about ***Nutritional therapy***? |  |  |  |  |  |
