# Supplementary material for: Iranian healthcare professionals’ knowledge, attitudes, and use of complementary and alternative medicine: a cross sectional study
Source: BMC Complement Med Ther. 2021 Sep 30;21:244. doi: 10.1186/s12906-021-03421-z (PMC8485522; doi:10.1186/s12906-021-03421-z)
Supplement: Supplementary file 4 — Additional file 4. Questionnaire of using of CAM modalities. [file 12906_2021_3421_MOESM4_ESM.docx]

**Additional files**

**File name:** *Additional file 4*

**Title:** *Questionnaire of using of CAM modalities*

- **Please express your opinion about using each of CAM modalities**

| **CAM modalities** | | **Daily** | **Weekly** | **Monthly** | **I use if necessary** | **I have not used it yet** |
| --- | --- | --- | --- | --- | --- | --- |
| **Mind and Body**  **Practices** | 1. Acupuncture |  |  |  |  |  |
|  | 1. Music therapy |  |  |  |  |  |
|  | 1. Energy therapy |  |  |  |  |  |
|  | 1. Hypnosis |  |  |  |  |  |
|  | 1. Massage therapy |  |  |  |  |  |
|  | 1. Magnetic therapy |  |  |  |  |  |
|  | 1. Meditation/Relaxation |  |  |  |  |  |
|  | 1. Yoga |  |  |  |  |  |
|  | 1. Exercise therapy |  |  |  |  |  |
|  | 1. Leech therapy |  |  |  |  |  |
|  | 1. Bloodletting |  |  |  |  |  |
|  | 1. Therapeutic touch |  |  |  |  |  |
|  | 1. Hydrotherapy |  |  |  |  |  |
|  | 1. Pressure therapy |  |  |  |  |  |
| **Natural**  **Products** | 1. Vitamin supplements |  |  |  |  |  |
|  | 1. Herbal medicine |  |  |  |  |  |
|  | 1. Nutritional therapy |  |  |  |  |  |
